# Supplementary material for: EnzML: multi-label prediction of enzyme classes using InterPro signatures
Source: BMC Bioinformatics. 2012 Apr 25;13:61. doi: 10.1186/1471-2105-13-61 (PMC3483700; doi:10.1186/1471-2105-13-61)
Supplement: Addtional file 5 — The Java code to format the data files, evaluate and predict. The file enzml_java_code.tar.gz contains the Java code used to format database data to ARFF and XML formats, to execute cross and train-test (jackknife) evaluations and to record evaluation results to database. More information is included in the readme.txt file and the Javadoc files. The code can be used with a MySQL database. To use a different database software, other JDBC drivers might be required. [file 1471-2105-13-61-S5.gz › java_code/utils/doc/uk/ac/ed/inf/utils/setutils/Set.html]

Set


---


|  |  |  |
| --- | --- | --- |
| **Overview** \| **Package** \| **Class** \| **Use** \| **Tree** \| **Deprecated** \| **Index** \| **Help** | |  |
| PREV CLASS   **NEXT CLASS** | **FRAMES**    **NO FRAMES**     **All Classes** |  |
| SUMMARY: NESTED \| FIELD \| CONSTR \| METHOD | DETAIL: FIELD \| CONSTR \| METHOD |  |


---


## uk.ac.ed.inf.utils.setutils Class Set

```
java.lang.Object
  uk.ac.ed.inf.utils.setutils.Set
```

**All Implemented Interfaces:**: java.lang.Comparable<Set>

---

``` public class Set extends java.lang.Object implements java.lang.Comparable<Set> ```

A set and its subsets. A set is a subset of a given set if all its elements
belong to the set. For example: a-b, or a-c, or b-d are subsets of a-b-c-d,
but a-e is not.

**Version:**
:   16 Mar 2011

**Author:**
:   Luna De Ferrari luna.deferrari-at-ed.ac.uk

---

**Constructor Summary**

`Set(java.lang.String id,
java.util.TreeSet<java.lang.String> elements,
SupersetsManager manager)`

`Set(java.lang.String id,
java.util.TreeSet<java.lang.String> elements,
SupersetsManager manager,
java.util.TreeSet<Set> subsets)`


| **Method Summary** | |
| --- | --- |
| `void` | `addSubset(Set newsetToAdd)` |
| `Set` | `clone()` |
| `int` | `compareTo(Set otherSet)` |
| `boolean` | `contains(Set set)` |
| `java.lang.String` | `elementsToString()` |
| `java.util.TreeSet<java.lang.String>` | `getElements()` |
| `int` | `getElementsSize()` |
| `java.lang.String` | `getId()` |
| `java.util.TreeSet<Set>` | `getSubsets()` |
| `int` | `getSubsetsSize()` |
| `java.lang.String` | `toReadableString()` |
| `java.lang.String` | `toString()` |

**Methods inherited from class java.lang.Object**

`equals, getClass, hashCode, notify, notifyAll, wait, wait, wait`

**Constructor Detail**

### Set

```
public Set(java.lang.String id,
           java.util.TreeSet<java.lang.String> elements,
           SupersetsManager manager)
```

---


### Set

```
public Set(java.lang.String id,
           java.util.TreeSet<java.lang.String> elements,
           SupersetsManager manager,
           java.util.TreeSet<Set> subsets)
```


**Method Detail**

### addSubset

```
public void addSubset(Set newsetToAdd)
```

---


### clone

```
public Set clone()
```

:   **Overrides:**: `clone` in class `java.lang.Object`

---


### compareTo

```
public int compareTo(Set otherSet)
```

:   **Specified by:**: `compareTo` in interface `java.lang.Comparable<Set>`

---


### contains

```
public boolean contains(Set set)
```

---


### elementsToString

```
public java.lang.String elementsToString()
```

---


### getElements

```
public java.util.TreeSet<java.lang.String> getElements()
```

---


### getElementsSize

```
public int getElementsSize()
```

---


### getId

```
public java.lang.String getId()
```

---


### getSubsets

```
public java.util.TreeSet<Set> getSubsets()
```

---


### getSubsetsSize

```
public int getSubsetsSize()
```

---


### toReadableString

```
public java.lang.String toReadableString()
```

---


### toString

```
public java.lang.String toString()
```

:   **Overrides:**: `toString` in class `java.lang.Object`


---


|  |  |  |
| --- | --- | --- |
| **Overview** \| **Package** \| **Class** \| **Use** \| **Tree** \| **Deprecated** \| **Index** \| **Help** | |  |
| PREV CLASS   **NEXT CLASS** | **FRAMES**    **NO FRAMES**     **All Classes** |  |
| SUMMARY: NESTED \| FIELD \| CONSTR \| METHOD | DETAIL: FIELD \| CONSTR \| METHOD |  |


---
